# Selections and Abstracts

**Published:** 1906-01

**Authors:** 


### SELECTIONS AND ABSTRACTS.

### THE DEBT THIS GENERATION OWES TO SURGERY. *

When a man is called to describe the charms of his sweetheart before a mixed company, some of whom know her well and others need enlightenment, he is either coy and silent or becomes enthusiastic, not to say maudlin. Since, through the kindness of your committee, I have been asked and have assumed the responsibility of speaking on the surgery of this generation, I must avoid the horns of the dilemma and speak circumspectly, not as fools, gladly, but as wise, redeeming the time. To acquit myself of arrogance at the outset, let me state at once that surgeons do not claim to be the only living live-savers. All learning is for our good, whether it result in practical, positive acquisition or the often equally beneficial negative of avoidance. The hand that rocks the cradle, that later wields the slipper or the birch, shapes our minds (as well as portions of our anatomy) toward judgment, equity and truth, and makes men of us, capable of discerning and applying theories and facts for individual and public good. So I claim that the mother of a Morton, the little minister of Lister, the gymnasium teacher of a Koch contributed in no small way to the sum of human happiness and relief, while scattered over the broad earth there are thousands who directly or indirectly have added their mite to the dower that Westinghouse or Morse or Guttenburg, life-savers in other fields than ours, have brought to the animal and human race. The very arch-enemy of life, the soldier, too, contributes, knowingly, or unknowingly, his pittance toward life-saving. The modern bullet is more merciful than its ancestor. It is intended to stop, not kill, and though this contention may seem answered by the rotting plains round Mukden, who can say but that from such top-dressing a race of Russians may arise worthy of freedom and its

* Read by Robert W. Johnson, A B., M.D., Professor Principles and Practice of Surgery, Baltimore Medical College, before the Medical and Chirurgical Faculty of Maryland, April 26, 1905.

privileges, or that the Japanese Sun will not let in the light of Asia, that will more than pay for this tremendous sacrifice. Having shown you that we are not the only claimants to philanthropy, let us now look at surgery, which, with medicine, does claim to make life-saving and relief its chief end and object. You may laugh in your sleeve at this when you hear, or personally know, of the extortionate fees sometimes charged, and think of it as a gilt-edged philanthropy. You may recall that the sign of the *Medici*, three balls or pills, is now the sign of the pawnshop, one of the sequelae of appendicitis, but it is nevertheless true that one of the greatest rewards and inducements of our profession is that we can make our living by saving lives and relieving pain, which, to my mind, is more of a vocation than cornering wheat or lard or advancing a cent on oil, though these occupations may be essential, too.

I will not drag your tired minds through all the halting steps of surgery, from Homer to Hippocrates, nor from Galen to the Hunters, an era when our science rose and fell like the ceaseless tides—one day its home among the temples with priestly devotees of Aesculapius, again sinking to a trade relegated to barbers and their ignorant apprentices. The very pole you see so often before the doors of "tonsorial parlors" is not an emblem of patriotism in its red, white, and blue stripes, but the sign of the cuppers and leechers, who used colored ribbons to wind around the arm of the sufferer whose blood was to flow until he fainted to satisfy the charlatan, who would thus free the system of the pernicious humor supposed to reside in what we know to be a life-giving stream. I will not fret your imagination by asking you to look on Marathon or Waterloo, nor attempt to paint the tortures of hot iron and boiling oil before the days when Paré (1564) insisted on the ligature, and thus immortalized his name, barber surgeon as he was, and one who believed in the oil of poppies as specific in gunshot wounds. Think of the strife between these ignorant philanthropists and their equally ignorant but more cruel competitors, the executioners. These latter men were supposed to have special dealings with the powers of evil, and in consequence to have special knowledge of the means of curing diseases considered to be due to witchcraft. A part of their business was to dislocate joints by the rack or to break bones upon the wheel, and hence it was supposed they had special skill in the repair of fractures and of dislocations. A Daniel come to judgment in the person of Frederick the Great of Prussia, as late as 1744, gave out this edict: "If the surgeons are ignoramuses, the public must not suffer, but must submit to be treated by the executioner rather than to remain lame or crippled" (Dennis' "System of Surgery," Vol. I, p. 77).

Let us not hark back, however, to those dark ages for contrasts. Surgery probably was on a par with the other sciences of that time when superstition and ignorance held sway in the human mind and men uninfluenced by Bacon's reasoning chose the *a priori* method of translating facts by theories and strained the facts rather than the theories to make both ends meet. Let us rather take a time which some of us can remember, a date that a father of seventy years may recall, just dimly perhaps, but a part of the generation now with us, and consequently cognate to my subject, say 1840, and let us see what was the condition of surgery at that time, and contrast it with that of 1905.

Take Colles' "Lectures on Surgery," published in 1845, and written by a man whose thirty-five years of professorship of surgery in Dublin gives him a fair title to be a typical surgeon of that date even if he had not immortalized his name with the most frequent fracture with which we have to deal. What do we find in his work? I am almost tempted to say about the same status as Marconi would find on the electrical information of the same date, except as to the mechanical or handiwork element of the craft. Thirty centuries look down on us here as they did on Napoleon, and yet the most striking features in the landscape are the pyramids, full of dead bones, and the ever-silent Sphinx. While wondering at the courage of our forbears, both to operate and suffer operations, and appreciating what effort and often self-sacrifice each little advance cost its author in ridicule and threats, yet we can truly say that surgery as we know it today was not yet born, but laboring to that end. The heroic McDowell had operated on the gallant Mrs. Crawford in 1809 in the face of a howling mob that threatened to lynch him if he failed, but, as a rule, the cavities of the body were still sealed to the scalpel, and opening them was like opening the box of precious ointment when the verdict was, "She hath done it for my burial." Take gunshot wounds of the abdomen, and what was the accepted treatment? "Bleed, bleed, bleed" is all you can do for him (Colles, p. 128). Reduce, reduce, reduce a man on the verge of collapse. Verily, except for its grim effect, a *reductio ad absurdum.* What had the writhing patient, then, to mitigate the terror of apprehension or the anguish of fulfillment? Opium. Thank God for that little. The lancet until a faint for a few short moments brought unconsciousness; emetics, tobacco, to relax like the first cigar, and many of you know how preferable death is to that aftermath, and then brute force, not cruelly, but cruel to hold down the shrieking child or best beloved, while the surgeon nerves himself for his ordeal, deaf to entreaties, curses, groans and prayers, does his well-intentioned work with celerity, if not care, and speed is more desirable than accuracy. Should the victim escape alive from the operating-room, new and ever-present dangers awaited him in the ward. So rife were erysipelas, hospital gangrene, blood-poisoning, tetanus, and the other powers of the air that many hospitals had to close their wards to prevent a total loss of surgical or obstetrical patients. Nor was this confined to hospital practice. Time after time men would lay down their private practice and leave the neighborhood to stop the train of dissolution that followed in their wakes. Mother after mother died from fever, and the doctor, in despair, thought himself responsible, and yet he knew no cause for it. These, too, were met by the lancet, salivation, and depletion until life left *per vias naturales.* But why dwell on the nightmare of science and lore doing their best under ignorance to lighten the darkness before the dawn, for "Now the day spring from on high *is* visiting us, and those that sat in darkness and the shadow of death, to them a light has sprung up." Nothing in all the domain of knowledge, nothing in all the workings of the human mind, no Homer, no Shakespeare, Milton, no man has done so much. The greatest God-given boon ever vouchsafed to humanity, without a rival except the Christ in His sermon on the mount, where love of man, the mainspring of all our work, was instilled in the human heart and philanthropy taught the fatherhood of God and the brotherhood of man, can excel the firstborn of modern surgery—anesthesia.

Sancho Panza says, "Blessed is the man that first invented sleep." It is no quixotic tribute to say that Long of Georgia in 1842 and Morton and Warren of New England in 1846 should be canonized, or that Simpson of Edinboro should have a halo round his memory. All honor, too, to that great Queen Victoria, who braved the actual dangers of chloroform as well as the threatened blight of narrow churchmen, shaking off the shackle of the curse of Eden and telling women everywhere that the edict "In sorrow shalt thou bring forth children," had been supplanted by the modern kindly-spoken order, "Breathe it in quietly."

Following soon after the discovery of anesthetics came another advance which, though apparently trifling and simple, had a great influence on wound treatment and its results. I refer to the introduction of the drainage-tube by Chassaignac. The drainage-tube was to the surgery of that date as important as aseptics is to ours, and even now in suppurating wounds that reach us after infection it is very essential. Esmarch's bandage, next to the ligature, has done more to check the flood of blood from wounds than any other invention, so that now hip-joint amputations cause no more hemorrhage than an ordinary nosebleed, and this generation is making up, by securing vessels before they are cut, for the bloodshed caused and advocated by our surgical and medical ancestors. In the latter part of the sixties twin stars appeared in the surgical horizon, hardly recognized at first, nebular in fact, but destined by their development to produce a tide, not twice a day, but perpetually, of advance and blessing—Pasteur and Lister, the French laboritorian and the Scotch clinician. Who has looked upon their faces and not been impressed with the intense zeal for honest inquiry and determination to find the truth shining out in the earnest, penetrating eye of the French chemist, and who has looked on Lister and spoken with him but feels intuitively, here is a most benignant man? A new science has sprung from their brains, not like Minerva, full-armed from Jove's, but step by step, line upon line, here a little and there a little, until modern surgery now stands refulgent before the world. Yet this was not all plain sailing.

Ignorance, jealousy, ridicule had to be met and overcome. Twenty five years ago Morrell McKenzie told me in London that Mr. Lister's plate on his front door had vitiated the price of property in the neighborhood. It is the old cry, "Away with Him," the benefactor; "Give us Barrabas," the robber. There is no way to estimate the value of antiseptic surgery. Ghosts that walked the wards have been laid in the banishment of sepsis, tetanus, secondary hemorrhage, erysipelas, puerperal fever, etc. The exploring scalpel now throws light on cavities previously open to the homicide alone. Diseases a generation ago guessed at and fatal are recognized and removed without pain or danger of inflammation, without the ever-dreaded daily dressing, without the disgusting sights and smells of foul wounds—only a line, hardly perceptible, marks the track of the surgeon. Surely antisepsis is a close second to anesthesia. To put this fact in figures, let me quote one item from a small hospital (180 beds) in a Western town where two brothers do the operating. Last year these Mayos operated on the abdomen 1,788 times, with the loss of about 3 per cent. of all cases, including cancer. Before Lister and Pasteur they would have done well to cure 3 per cent., with 97 deaths out of every hundred, if they themselves had escaped hanging. The abdomen is not the only cavity open to inspection and treatment through modern surgery. Thanks to the researches of such men as Broca, Goltz, Fritsch, Hitzig, Ferrier, Horsley, Keen, and others, the brain, this *sanctum sanctorum*, has yielded its secrets. We are able to diagnose, and what is far better, to treat with a moderate degree of certainty, abcess, tumor, hemorrhage, pressure, and effusion, not with the witchhazel wand of the charlatan or the trephine of the Aztec, who made his epileptic patient look like a human pepper pot, but definitely marking out the boundaries of the trouble before the skull is entered, and going down directly over the spot involved. From the little cloud no bigger than a hand, we can, like Elijah of old, foretell the storm that is brewing round the capital, and, like Franklin, divert the lightning stroke of death, or, with Röntgen, use that very lightning to discern or dispel the cause. Cocaine, too, belongs to this generation. Time forbids that I should ask you to follow the discoveries in special departments of surgery, each one a volume. How eager to advance, it knows no jealousy, and adopts for its beneficent purposes the laryngoscope of Manual Garcia, the music teacher, with the same avidity as the ophthalmoscope of the surgeon-physicist, Helmholtz, or Sims' speculum, the cornerstone of gynecology. Dr. Holmes tells us how medicine profited by other inventions and discoveries. From a monk it got antimony, from a Jesuit how to cure agues, from a friar how to cut for stone, from a soldier how to treat gout, from a postmaster how to sound the Eustachian tube, from a dairyman how to prevent smallpox, from an old market woman how to catch the itch insect. Nor does it claim from its sister, medicine, more than it is willing to give in return. If we have laid our hand on the appendix, we have given back diphtheria, and nowhere will you find more earnest advocates of serum therapy than surgeons, who are glad to surrender the scalpel to the Pravaz syringe. Last, but not least, you of this generation, who are born free, enjoy not only the results of investigation, but are permitted, if you so desire, to take part in it. Hundreds of great surgical questions still await the spear of Uriel. A thousand rocks on which science splits need but the proper Moses to strike and bring forth the pure water of truth and helpfulness. You who are not fettered by superstition or trammeled by dogmas of the Middle Ages, rejoice in your freedom! Realize your responsibility! Quit yourselves like men!

---

### THE TREATMENT OF BRONCHITIS IN CHILDREN.

J. E. Winters in *Medical News*, November 25th, recommends the following treatment:

Clinical experience and pathological investigation yield ample proof that bronchitis in a young child is never slight.

The coryza of today may before the morrow spread to the small bronchi, for, like its congener, catarrhal croup, its onslaught is commonly at night. To forestall and thwart extension is the pith of treatment. If this is accomplished fatal issue is averted. This is well-nigh attainable in every instance.

Distention of the branches of the bronchial arteries is the first deviation from the normal. The design must be to restrain and counteract this at its inception.

By the cutaneous capillaries, by the intestinal mucous membrane, and by cardiac inhibition, the initial lesion may be subdued and abridged; irritation of the mucous membrane may be abated by the air the patient breathes—mild diaphoresis, gentle catharsis, cardiac sedatives, warm soothing air for the irritated respiratory surface.

The temperature of the respired air should be uniformly 72° F.—day and night—sun exposure and open fire if practicable. Ventilation must secure at all hours pure, fresh air.

The child should be in a crib—never on a bed. The crib should be flannel-lined and be in the center of the room—never near a wall, window or door. Sometimes it is necessary to place screens about the crib to avoid draught.

Light flannel should envelop the child's body, arms and legs. The flannel shirt should be secured to the diaper by safety-pins, back, sides and front; long worsted stockings should be worn and likewise fastened to the diaper. The wrap and shirt must be loose and open in front, secured by tapes or safety-pins, assuring ready access for physical examination. A duplicate suit is to be kept in readiness. A quick hot bath precedes flannel envelopment.

In a case of moderate severity nine-tenths of the treatment has been compassed.

DRUGS.

In severe cases the drug of unfailing, universal efficacy, is aconite. The bronchial arteries, branches of the aorta or intercostal arteries, subject to direct, immediate, forcible cardiac pressure, surge with blood. To restrain and limit this is the aim. Through this drug arterial pressure is promptly circumscribed. Maximum, frequent doses during the first hours; diminished, less frequent doses after four or six hours; early abandonment. It should be given in water only—tasteless, non-nauseating, it does not affect appetite. For a child of one year,

℞ Tr. aconite (Fleming)................................gtt iv
Aq. destillat......................................ounces iii

M. Sig. One teaspoonful every fifteen minutes for one hour; every half hour for four or six hours; then every hour for twenty-four more hours.

Turgescence ceasing the remedy is discontinued.

Arterial pressure is lessened by diaphoresis. Sweet spirits of niter the pre-eminent diaphoretic. Niter, citrate of potassium, and spirits mindererus may alone be used where aconite is not urgently indicated, and follows discontinuance of it. The combination often nauseates, and even more frequently affects the sense of taste so disagreeably that nourishment is persistently refused.

Intestinal elimination, diaphoresis, aconite and niter make up the febrifuge measures. Other antifebriles (baths, sponging, coal-tar derivatives) are, individually and collectively, contraindicated. Cold to the cutaneous capillaries is unphysiological, pernicious; conduces to extension.

STAGE OF SECRETION.

Excessive secretion may inundate the bronchi and must be anticipated and intercepted.

The agents which diminish secretion are camphor, carbonate of ammonia, nux vomica, oxygen inhalations and counter-irritation. Sprits of camphor is the most valuable drug agent.

For a child of one year,

℞ Spirits camph. ............................................ ʒi
Saccharin........................................................gr.i
Spts. etheris nit. (for preventing precipitation of camph.) ʒii
Syrp. tolut. (for diminishing pungency)............... ℥ss
Aq. gaulth. q.s. *a. d.*.......................................... ℥iii

M. Sig. One teaspoonful every half hour.

Carbonate of ammonia in one grain doses is a valuable adjuvant to this mixture, but often provokes nausea. Nux vomica has the same objection.

When the tubes are loaded with tenacious secretion, mustard is of priceless service. One part mustard, six parts flour mixed with *cold* water and white of egg, and applied as a paste between two layers of thin linen, covering the entire region where moist sounds are heard, left on twenty to thirty minutes and renewed every two or four hours, according to the condition of the skin.

Where these resources have not been initiated at the opportune moment, or a child has not been seen until the bronchi are flooded with secretions, superinducing laborious and yet shallow breathing; on auscultation very few râles or none at all are heard, breath-

4

sound absent over both bases posteriorly; pulse exceedingly rapid and small and disappears under the finger; suppressed cough from $CO_2$, medical art is put to its surest test, for even this extremity is surmountable by skilful adaptation of that knowledge and power born of pathology and experience.

Impetuous stimulation is the common, almost universal blunder. The deluged tubes must be unloaded, emptied; respiration and oxygenation re-established. Blocked bronchi, soft, yielding thoracic walls; weak, exhausted inspiratory muscles, make lobular collapse irresistible. The only redeemable, relievable factor is the obstruction to the passage of oxygen.

Witness the breathing become more and more shallow, and there is only slight movement of a few ribs and faint jerking contractions of the diaphragm; every untoward phase is accentuated swiftly. The swallowed sputa produces vomiting: The diaphragm, the abdominal muscles, the muscles of the lower part of the chest compress the thorax as in a vise. The tubes are squeezed, the contents forced out, mucopus wells out of the mouth. A sudden deep inspiration, the whole chest expands, the breath-sound is heard over the entire pulmonary area. Air has penetrated the most remote air-sacs; oxygenation is renewed, blood-cells revived, reanimated, cyanosis is replaced by vivid pink!

The position the child was in while vomiting—face downward—is maintained, the secretions gravitating to larger, sensitive bronchi, induce cough and are ejected instead of being swallowed. The cue is obvious—emetics, postural treatment.

When the air tubes are blocked by tenacious mucopus they can only be forced by active emesis. This should not be repeated more than once or twice in the twenty-four hours. Two or three clearings are adequate. The child must be kept in the prone position to secure gravitation of secretions to a sensitive mucous surface to avoid refilling. Oxygen inhalation must be *continuous* in this critical state—even during sleep.

The forceful resources are supplemented by mustard packs and maximum doses of spirits camphor and carbonate of ammonia.

Steam inhalation is condemned, as it is not only useless, but hurtful.

### THE MALADIES OF WOMEN—UTERINE AND NERVOUS.

BY W. C. BUCKLEY, M.D., PHILADELPHIA, PA.

Case from my notes—Miss R., aged 20, had suffered for several years. She complained of digestive disturbances, diarrhea, vomiting, headache—mostly in the back part of the head. The head pain often began in the forehead, and, as was determined later, was due to myopia and astigmatism, which were corrected. I treated her for a year for these and other troubles, by the administration of the arsenates of quinine and strychnine, together with the "uterine tonic" pill. She now very seldom complains of any of her old symptoms, which no doubt were occasioned by nervous disturbance, rather than any organic disease. The nervous system, no doubt, contributes greatly to the suffering of women. But how? Let Dr. T. C. Allbutt answer. "How intimately," he says, in speaking of the uterus, "this organ, or this system—that is, uterine appendages—is associated with the nervous system is well known. But unfortunately the weight of knowledge leans one way—it leans to a curious and busy search for every local ill which may arise in the female pelvis, while blind oblivion scatters the poppy over every outer evil which, in its turn, might hurt the uterus; nay, even more, a resolute prejudice would deny that, in the woman, any distress can arise which owes not its origin to these mischievous parts. . . . "The uterus has its maladies of local origin, its maladies of nervous causation, and its maladies of mixed causation, as other organs have; to assume, as is constantly assumed, that all uterine neuroses, or even all general neuroses in women are due to coarse changes in the womb itself, is as dull as to suppose that the stomach can never be the seat of pain except it be the seat of some local affection, or that the face can never be the seat of *tic-doloreux* unless there be decayed teeth in the jaw. All mucous membranes, indeed, seem to betray nervous suffering by relaxation—and its opposite, especially of the blood-vessels connected with it—or changed secretions, and I make no doubt whatever, that a large number of uterine disorders which are elevated to the place and name of disease of the uterine system are but manifestations of neuroses."

All neuroses are more common in women than men, especially facial neuralgia and migraine, also gastralgia and pseudo-angina. Not only so, but they possess one more organ with its own nervous connections and its own added chapter of diseases and neuroses. But to say that all these maladies are due primarily to uterine diseases is to talk wide of all analogies. "Women, speaking generally, feel pain more than men do; patient as they are, they seem to have less reserve of force and less resistance, more susceptibility and resentment, and less capacity. Yet there is no standard of pain, nor of men, by which you shall say this patient is a coward and his outcry exaggerated. Men and women are variously organized in respect of resistance of pain, and their fortitude or despair must be tested, not by their cries, but by other features of their character."

The study of temperament in these cases will reveal much for the physician in diagnosis, prognosis and treatment. Lymphatic people are generally the subjects of excessive *spinal* innervation, they are bold, aggressive, and enduring in character and suffer less from pain. Nervo-bilious subjects are inclined to suffer from excessive *ganglionic* innervation; these crouch with fear, from slight causes; always inclined to look behind them in fear of harm. The honest and fair-minded man or woman with the courage of his convictions present ordinarily the opposite temperament. These phenomena are to be explained by the connections of the reflex nervous mechanism with the sensori motor centres of the sensorium. The temperament that shows mildness, sweetness, gentleness, that is afraid to hurt others in that it loves to do good, is the one that bears toil and endures pain. On the other hand, the temperament of impatience, impetuosity, of love of self, is of the opposite kind. Those of the first class are easily cured of their diseases, comparatively speaking. Gelsemium, a *ganglionic sedative,* is well adapted to a large majority of pathological conditions, and mainly those that depend primarily upon derangement in the ganglionic nervous system, and secondarily, upon derangement of the vascular system by which passive congestions are produced. In such instances the above remedy must be given in small doses; for active congestion, which occurs mostly from excessive spinal or cerebro-spinal innervation, hyoscyamine and atropine serve as types of remedy to use in this class of cases.

Among the former ailments those resulting from passive congestion, the active principle of gelsemium may be employed most effectually. In diseases of an emotional character, the effects of grief and other nervous disturbances, the pulse often becomes feeble, respirations become irregular, with occasional deep sighing. If the shock has been profound other functional troubles are likely to follow, for example, muscular weakness, stomach and liver disorder, with either weak or confused vision. Dull headache involving the forehead usually follows, though the occiput may be the centre of pain. Here strychnine, or strychnine hypophos., may be alternated with the gelsemine. The dosimetric granules of these remedies are most suitable, because they are prompt and reliable. Sunstroke, vertigo, meningitis, cerebral vascular disturbances more or less severe, all call for gelsemine and strychnine, combined with the free use of the saline laxative. —*Southern Clinic.*

---

### THE PANAMA CANAL.

Dr. Victor C. Vaughn (*Jour. A. M. A.*, Sept. 9th) offers the following brief statements which represent his views concerning the means that should be adopted in carrying out this great enterprise:

1. The first thing that should be done is to render the Canal Zone a habitable place. That this has not been done, although we have been in possession for more than two years, must be admitted by all.

2. The engineering problem involved in the construction of the Panama canal is of secondary importance compared with the sanitary problem which must be met.

3. The French failed to complete the canal, not because they had not the engineering ability necessary to do it, but because they were wholly unable to cope with the insanitary conditions existing in that locality.

4. In converting the Canal Zone into a habitable location, engineering skill must be employed, but it will be only a secondary and subordinate part of the work that must be done in accomplishing this great task.

5. In rendering the Canal Zone habitable sanitary authority should be supreme and should control and direct all other agencies.

6. A thoroughly equipped scientific laboratory, provided with the best obtainable in the way of apparatus and men, should be provided and put into operation on the Isthmus of Panama.

7 The work so auspiciously begun by the lamented Reed and his colleagues should be continued.

8. The number of men employed on the isthmus during its sanitary development should be as small as is consistent with efficiency.

9. These men should be protected from the mosquito by mechanical devices applied to the individual as a part of his dress, or to a small group of men in the form of areas covered by mosquito bars.

10. During the sanitary development travel between this country and the isthmus should be as nearly as possible confined to the winter months, and should at all times be restricted and closely guarded in every scientific way in order to prevent the importation of infection into the United States.

11. Expert sanitary workers, together with expert entomologists, should be employed in the scientific laboratory and should be given every facility which their investigations may demand.

12. The mosquito should be subjected to the closest inspection with the purpose of securing its extinction.

13. The conditions under which the mosquito breeds and the effect of insecticides on it at this and other times should be carefully and scientifically investigated.

14. The parasites that infest the mosquito should be studied and, if found desirable, be scattered all over the isthmus. In this connection I wish to state that Dr. Lamb, of Owosso, Mich., who served some years with the army in the Philippines, has informed me that there is a louse that feeds on certain mosquitoes and destroys them in a few hours. Whether this could be used in the extermination of the stegomyia or not can be determined only by experiment and observation.

15. Vaccination with modified virus of yellow fever should be studied most thoroughly, because in this direction there is the possibility of securing immunity to the disease.

Certainly the canal can be built without attention to sanitation, but if the method followed up to the present time be continued it will cost thousands of lives, and, as the present epidemic in the Southern States shows, it will, with greater or less frequency, extend to this country and involve greater or less areas.

An attempt to dig the Panama canal is like stirring up a nest of poisonous hornets, only that the comparison is a weak one. In this case the disturbed poisonous insects must get their poison from man and, having done this, they may pursue their foe even to the distant parts of the earth. Shall we continue to feebly whip the wasp's nest and drive the vicious insects abroad, or shall we wait until all are quietly in their house and then destroy them before they can do us harm? The former is the "strenuous," the latter the sensible way to do it.

---

### NIHILISM IN THERAPEUTICS.

The skeptic voices his doubts somewhat as follows: "Specifics you can count on your fingers—quinine, mercury, the iodides, antidiphtheritic serum, the salicylates probably, the bromides perhaps, iron and arsenic it may be—and you're done. A handful of diseases directly controllable, but the overwhelmingly greater number beyond our power either to govern or to stay. A diagnosis, some experimental drug giving—for every line of treatment is an experiment, just as every diagnosis is a guess—a recovery ascribable rather to Nature than to drugs, or possibly a confirmatory autopsy; such is the inglorious role of medicine." This view of our limitations is pretty general, something of a fashionable intellectual pose, particularly among the younger ultrascientific men; and it must be reckoned with.

It may be replied, and the oftener the better, that, though we have few specifics for diseases, we have *many for symptoms.* The most superficial view will remind the cynic that we are virtually in control of such general manifestations as pain, hyperpyrexia excess and deficiency of vascular tension, cardiac weakness or overaction, dropsies, sleeplessness, cough, constipation, diarrhea, excessive sweating, vomiting, delirium, and a host of lesser bothers; so that, while we can not always stop the disease, we can inhibit its phenomena, which, unrelieved, may alone and of themselves cause a fatal issue.

No analogy more nearly walks on all fours than the comparison of a patient in the grip of, say, a continued fever and a ship in a storm. No power can stop the storm, but much can be done to help the staggering vessel ride it out. And it is as reasonable to disparage the officer's services on the bridge as the doctor's at the bedside. There is art as well as science in both seamanship and medicine. The skilled master does a hundred things with sails and helm which ease the straining bark, but are not taught in any treatise on navigation; and the veteran practitioner intuitively meets danger by combinations not found in any pharmacology. And his instinct directs equally what should be left undone—when to stand by with all hands, nor touch ever a brace or a halyard, while the good ship glides past the reefs into the harbor.

There is the sense of that quip which stings a bit in the ear: "There is much difference 'twixt a good doctor and a bad one, but little 'twixt a good doctor and no doctor at all." How that truth smites the cosultant when he finds some typhoid case laboring like a vessel in distress under the use of salol and bismuth and strychnine and quinine and quinine and turpentine and alcohol, when it would ride lightly under the orders given nearly a hundred years ago by grand old Nathan Smith: "He fed the patient largely on milk, he gave him to drink copiously of clear water, he stimulated him at times, he withheld strong drugs, he kept him in a cool, well-ventilated room, and he drenched him frequently with cold water when the fever ran high." (Mumford, *Medicine in America.*) There is a time to give and a time to withhold, but cut and dried science fails to teach us when. In practice the brain's laboratory will never be supplanted by the pathological laboratory. Not every craft weathers the gale. Resistless violence, unsuspected currents, hidden weaknesses wreck, spite of science and skill. Nihilists keep repeating that but little variation of results in pneumonia is shown by statistics of whatever form of treatment. True, and similarly, Lloyds' reports a pretty even average of marine losses from year to year. But for that reason will you venture to sea without a captain?—*Editorial New York Med. Jour., Phil. Med. Jour., Dec. 2, 1905.*

---

### SPREAD OF TUBERCULOSIS.

At a meeting of the Baltimore County Teachers' Institute, in discussing the Spread of Tuberculosis, Dr. G. Milton Linthicum said in part:

"I believe if all children could be properly instructed in regard to tuberculosis and would make an effort to carry out these sanitary laws that in thirty years the mortality from consumption would be reduced more than half and in a reasonable time eradicated from the earth.

"It is of great interest and special importance relative to tuberculosis to note the growth of the lungs during school life. At nine years a boy's vital capacity—the greatest amount of air that may be expelled from the lungs after the deepest inspiratory effort—is 1,500 cubic centimeters, which gradually increases until twenty-one years of age, when its usual maximum of 4,221 cubic centimeters is reached. The greatest increase for any year is at the age of fourteen for boys and twelve for girls.

"Relative to the various diseases of childhood there is a remarkable lack of knowledge. Dr. Hertel examined 3,141 children in Copenhagen, and found 1,900 healthy, 978 sickly, 236 uncertain; 18 per cent. were sickly on entering school; this increased in two years to 30 per cent., and just before puberty had reached 40 per cent., when it dropped to 30 per cent., where it remained for several years. Of 1,211 girls between the ages of five and sixteen, 640 were healthy, 433 sickly, the rest not returned. The percentage of sickness for girls rose from the third year from 12 to 32 per cent.; from 12 to 16 sickness increased to such an extent that the sick outnumbered the well by 10 per cent., except at 14, when there was a slight betterment.

"It is a matter of great interest now, and will be a source of pride to us in future years, to know that Baltimore held the first tuberculosis exhibit, and at this exhibit resolutions were passed which resulted a year later in the formation of the American Association for the Prevention of Tuberculosis, having members in almost every State in the Union.

"Children should be taught in a simple way the truths relative to consumption—that it is dangerous to kiss a tubercular subject in the mouth; that they should never cough or sneeze without a piece of cloth or handkerchief before their mouth and nose, unless in the open air, and they should turn their heads away from anyone who might be near them. They should never spit on the floor; never swap apple cores or bits of candy which have come in contact with their neighbors' mouths, never turn leaves of books with wetted fingers, never put anything in the mouth except food and drink.

"I wish to warn you against phthisiphobia, a popular disease which has come into existence since the warfare against consumption has taken on renewed life. It means a fear of consumption; it means that the laity have gotten into their minds that consumption is contagious as is smallpox, namely, by contact. Teach children the truth relative to it, as it never does much harm, and unless this absurd notion is checked it will work great hardship upon the unfortunate sick."

---

### SCABIES.

Discussing the treatment of scabies, E. O. Huntington, M.D. (*N. Y. Medical Journal* and *Phil. Medical Journal*, December 9, 1905), says:

Successful treatment of scabies demands destruction of the parasites and cure of the dermatitis caused by them. The first of these indications is made comparatively easy to meet by the habit, which the acarus exhibits, of infecting those parts of the skin which are thinnest or least hardened, as between the fingers; the flexor surfaces of the wrists, the axillæ, and genitals, and where the burrows are easily opened to permit direct application of the parasiticide.

To accomplish this, my routine treatment is as follows: The patient is directed to take a warm bath, lasting about half an hour, during which time the infected areas are to be gently scrubbed with sapolio and a hand brush. Green soap and chalk or powdered pumice may be used for this purpose, but I have found sapolio convenient and eminently satisfactory in opening the burrows to expose the parasites.

Sulphur ointment is the parasiticide, par excellence, for the acarus. I use it alone or combined with balsam of Peru, the amount of the balsam being in direct proportion to the severity of the dermatitis. When pustular lesions are present, I touch them with liquefied carbolic acid and neutralize this with alcohol, before applying the ointment.

The patient is directed to rub in the ointment very thoroughly night and morning, for three or four days, which is the time necessary to cure the ordinary case. He should then take another warm bath and put on clean clothing throughout. This clean clothing should have been previously sterilized as a precaution against reinfection. If the dermatitis is not already cured by the removal of its cause, it will now readily yield to the application of any bland ointment or dusting powder.

To prevent reinfection of the patient and infection of others, all clothing and bedding used by the patient during the time of his infection and treatment must be disinfected. This is best accomplished by boiling such articles as will not be injured thereby, and subjecting the remainder to sterilization by dry heat. To recapitulate:

The outline of the treatment which I follow in a typical case is: 1. Warm bath for one-half hour, scrubbing infected areas with sapolio. 2. Carbolic acid and alcohol applied to pustules. 3. Sulphur ointment, alone or with balsam of Peru, applied twice daily for three or four days. 4. Warm bath, followed by putting on complete outfit of clean clothing. 5. Disinfection of clothing and bedding used by patient during infection and treatment, by boiling or dry heat. 6. Bland ointment or dusting powder, if dermatitis persists.

### AN INTERESTING ANALYSIS.

Chemical Laboratory,
H. ENDELMANN, PH.D.,
Analyses, Consultations and Researches.
23 William Street.

NEW YORK, N. Y., November 7, 1904.

### CERTIFICATE OF ANALYSIS.

I have examined the sample of Liquozone bought from Schieffelin & Co., marked as stated below and find the following results:

MARK OF SAMPLE. Manufacturers, Liquid Ozone Co., Chicago, Ill.

| | |
|---|---|
| Specific gravity | 1.0113 |
| Reaction strongly acid. | |
| Residue at 100° C. from 100 parts (liquid) | 2.012 |
| ash | 0.035 |

Detailed analysis: 100 parts by volume contain substances by weight as follows:

| | |
|---|---|
| Water and volatile substances at 100° C | 98.375 |
| Free sulphuric acid ($H_2SO_4$) | 1.418 |
| Sulphate of iron | 0.014 |
| Sulphate of alkalies and earths | 0.035 |
| Sulphurous acid ($SO_2$) | 0.114 |
| Hydrochloric acid | 0.044 |
| Traces of ammonia, silica, etc. | |

The oxygen required to oxidize the organic matter is 0.0613. This is mainly sugar or saccharine matter, and such material as may be extracted from wood.

H. ENDEMANN, PH.D.

Sworn to before me this — day of November, 1904.

H. SCHAAF,
Notary Public New York County.

DR. J. C. MIMS,

Analytical and Consulting Chemist,

Chemist to the Board of Health.

825 Gravier St.

ANALYSIS OF

Fertilizers,

Cottonseed Products,

Waters, Minerals, Etc.

NEW ORLEANS, LA., November 10, 1904.

Analysis No. 12412.

### REPORT OF ANALYSIS OF A SAMPLE OF LIQUOZONE.

Reaction, very strongly acid.
Specific gravity, 1.0074 at 17.5° C.
Color, pale amber.
Odor that of sulphurous acid.

| 100 c. c. contains: | grams. |
| --- | --- |
| Sulphuric acid | 0.8360 |
| Hydrobromic acid | 0.0451 |
| Sulphurous acid | 0.2826 |
| Silica and silicates | 0.0051 |
| Oxides of iron and aluminum | 0.0023 |
| Oxides of calcium and magnesium | 1.0016 |
| Alkalies | 0.0049 |
| Organic matter | traces. |
| Total residue at 100° C | 1.1861 |
| Water and volatile matter | 98.8139 |
| Available oxygen | none. |
| Ozone | none. |

JOHN C. MIMS,
Analytical Chemist.

STATE OF LOUISIANA, }

} SS.

PARISH OF ORLEANS. } City of New Orleans.

On this tenth day of November, 1904, personally came and appeared before me the undersigned authority, John C. Mims, to me well known who, being duly sworn, deposes and says that the above and foregoing is a true report of his analysis of a sample of Liquozone purchased by him in the open market.

This 10th day of November, 1904.

C. G. GILL, Notary Public.

---

THE following prophylactic measures for the prevention of pneumonia are suggested by Dr. Edward F. Wells, of Chicago: (1) Pneumonia sputum should be destroyed before it has become dry. The sputum which clings to the teeth and lips, and that which may adhere to the fingers and bedding, should be wiped up with moistened cotton, gauze, or other cloth and these burned. All sputum, although not pneumonic, might well be destroyed. In coughing, in the case of the healthy as well as the pneumonic, a cloth, preferably moist, should be held before the mouth in such a manner as to prevent the projection into the surrounding air of the fine, insensible, but probably pneumococci-bearing spray which follows ordinary coughing. The same applies to sneezing and blowing the nose. (2) The nostrils, mouth, and throat should be kept as clean as possible. Cleansing sprays and washes for the nostrils; washes for the mouth, and brushing the teeth; gargles and drinks for the throat. Permanent occlusions of the nostrils should be removed, while transient ones may well be cared for by the more or less frequent use of a spray of adrenalin solution. Honey-combed tonsils and adenoid growths shonld be removed. Taking a drink of water after eating is a habit which should be formed and cultivated. Every means should be taken to prevent sleeping with the mouth open. (3) The room occupied by a pneumonic patient should be afterward disinfected, as is done after diphtheria and some other infectious diseases. Remaining long or unnecessarily in a room occupied by a pneumonic patient should be avoided. (4) Respiratory catarrhs from whatever infection should be avoided, and if contracted should be relieved as quickly as possible. (5) Physical and mental exhaustion, privation, undue exposure to cold and inclement weather, all should be avoided as far as practicable.

---

### VIRTUE RUNNING WILD.

The sentiment which underlies the present efforts of certain worthy medical men to protect the profession from imposition and to make our therapy clean, reliable and trustworthy, is entirely laudable and commendable. The extent to which some of these gentlemen are permitting their enthusiasm to carry them is lamentable. The judgment passed upon many of the pharmaceutical preparations which have stood the test of time for years in the practices of thousands of successful medical men has seemed hasty and ill-advised. To one who is prejudiced in neither direction, who endeavors to look at the matter with perfect fairness, it is very questionable if it is right that a small faction of the American Medical Association should use the organ owned by all of the members to condemn or detract from the reputation of long-established pharmaceutical preparations, many of which are used regularly by a large part of the membership of the Association. The manufacture and sale of pharmaceutical preparations is and must be commercial in its character. It can never be professional. The average preparation which has been used by medical men of intelligence for years with good results must have something in its favor, even if its manufacturers are not willing to conduct their business exactly as we may wish to dictate. I have no desire to uphold in any way the secret medical nostrum, but I question as a matter of fairness, the propriety of attacking any well-tried preparation until it is demonstrated beyond reasonable doubt that the members of the Association are opposed to, rather than being users of the preparation in question. Those who have been placed in positions of power—which may be used for the accomplishment of evil as well as good—should appreciate that such an office is one of trust and there should be an effort to carry out the will and wish of the majority rather than to be led by personal prejudice or petty motives. G. T. F.—*The Chicago Clinic and Pure Water Journal.*

---

### RENAL DECAPSULATION FOR NEPHRITIS.

Henry Harris, M.D. (*Johns Hopkins Bulletin*, December, 1905), reports a case of renal decapsulation for nephritis from which he deducts the following conclusions:

A patient with grave chronic parenchymatous nephritis after being treated energetically by medical measures in the hospital for two and one-half months, was discharged much improved. This improvement was only of short duration. One and one-half months after being discharged patient's condition was again serious and it was necessary to readmit him to the medical ward.

His second stay lasted only two weeks; at the end of that time his condition was very slightly improved. One month after this he entered the hospital for the third time, in a serious condition. At the end of a month he had not improved. Bilateral renal decapsulation was then performed. Improvement was noted about three weeks after the operation. In contrast to that of previous admissions, the improvement following operation has been permanent, now lasting sixteen and one-half months and has been very positive.

This improvement is seen both in the patient himself and in his urine. The daily output of urine has increased, the urea has increased. Albumin is present only in traces, and casts are only exceptionally found. Renal decapsulation has been of value in the treatment of this case.

Since writing these notes it is learned that the patient has sailed to Canton, China, in order to take the governmental examinations for a scholarship in this country.

---

### SITUS TRANSVERSUS AND ATRESIA OF THE PYLORUS.

H. M. Little (*Johns Hopk. Hosp. Bull. July, 1905*) reports a case of a newly-born child suffering with atresia of the pylorus. On account of the obstruction a gastro-enterostomy was performed on the sixth day, but the child died fourteen hours after the operation. The diagnosis of complete obstruction of the pylorus or upper portion of the duodenum was based on the following considerations: (1) The vomiting of a large amount of amniotic fluid shortly after birth; (2) recurrence of vomiting not regularly after the ingestion of small amounts of fluids, but as soon as approximately 20 to 30 c.c. has been given; (3) the presence in the stomach of the entire amount of a feeding (15-20 c.c.) when the stomach was washed out two hours after its ingestion; (4) the absence of bile-staining of the vomitus or fluid obtained at lavage; (5) the presence and passing of meconium gradually showing more and more the appearance of bile; (6) the absence of any sign of digested milk in the stool as late as the seventh day; (7) the uniformly subnormal character of the temperature; (8) the persistent loss of weight; (9) the anuria; (10) the fact that the mother had hydramnios. At autopsy there was found complete transposition of the thoracic and abdominal organs. The stomach showed that the pyloric end ended abruptly behind and to the left of the head of the pancreas.

---

BATTLE & CO., of St. Louis, have just issued the 8th of their series of twelve illustrations, of the Intestinal Parasites, and will send them free, to the physicians, on application.

---

An amputation for malignant ulceration should not be performed until the possibility of its being merely a broken-down gumma has been satisfactorily excluded.—*American Journal of Surgery.*

### FRONTAL SINUSITIS.

Thomson reports two fatal cases of frontal sinusitis, from which he draws the following conclusions: 1. In cases of multisinusitis it is well to drain the maxillary cavity some time before the frontal is operated on. Both cavities may be operated on at the same time; but if only one sinus is operated on at a time it should be the frontal sinus, the lower being drained until it can be opened. 2. In spite of free opening of the frontal sinus, the establishment of a large communication with the nose, and the avoidance of closure of the external wound a slow infection of the bone may take place leading ultimately to infection of the meninges. This may even be started in suppurating cavities on the opposite side to the one operated on. 3. The local condition of the wound, as well as the pulse, temperature, and feelings of the patient may fail to indicate the onset of mischief. After one to three weeks this is revealed by headache, pain, tenderness, and puffy swelling on the forehead or around the eyes. 4. When septic osteomyelitis has started the most vigorous measures may fail to arrest it. It may last one and a half years before terminating fatally. 5. The chief danger appears to lie in the ethmoid labyrinth, owing to its anatomical irregularities and to the difficulty of treating them satisfactorily. 6. Up to the present the operation which best meets these difficulties is that of Killian. In many cases a preliminary intranasal operation on the ethmoid is advantageous.—*Lancet, August, '05.*

---

### SUDDEN DEATH DURING OR IMMEDIATELY AFTER THE TERMINATION OF PREGNANCY OR OPERATION ON THE PELVIC ORGANS IN WOMEN.

E. P. Davis (*St. Louis Medical Review*, June 17, 1905), from a study of twenty-five cases, arrives at the following conclusions:

1. That sudden death may occur after abortion, labor or operation from undemonstrable causes.

2. Death may follow abortion, labor or operation, from the rapid formation of a pulmonary embolus. Pulmonary embolism may develop before the placenta has been delivered, or when delivered by Crede's method. It is not frequent after Cæsarean operations or after the more complicated obstetrical operations. Predisposing and probably exciting causes are bronchial infection and abnormal conditions of the blood.

3. Primary thrombosis and secondary embolism may cause death after the termination of pregnancy or operation. Causes: Pre-existing infection, mechanical violence, altered conditions of blood serum, and lesions in the walls of the valves.

4. Sudden death occurs in parturient women and after operation from reflex nervous shock without assignable cause; also from disease of the thoracic viscera, from infection of the placental site and other obscure conditions.

---

### THE RELATION OF THE APPENDIX TO PELVIC DISEASE.

Peterson presents the following conclusions from two series of clinical and microscopical studies:

1. In the first series 50 per cent. of the specimens were microscopically normal. In the second series 49.3 per cent. were also normal.

2. In the remaining 50 per cent. there was evidence of present or past acute or chronic inflammation.

3. The average length of the appendix was eight to ten centimetres.

4. It was adherent in 18 per cent. of the first series and 23.4 of the second.

5. Appendices may be club-shaped, constricted, or bent, and still be perfectly normal.

6. There were fæcal concretions in 8 per cent. in the first series and 16.4 in the second. The concretion did not always denote disease.

7. In seventeen cases, with chronic disease of the appendages, there was also disease of the appendix.

8. In some of the cases of chronic disease of the appendages the appendix, though adherent, was normal in other respects.

9. About half the cases in which there were uterine fibromata there was evidence of present or past disease of the appendix.

10. Of eight cases of ovarian cyst, nine were accompanied by disease of the appendix.—*American Jour. Obstetrics*, *August 1905.*

---

### INFLAMMATORY CONDITIONS OF THE APPENDIX ACCIDENTALLY BROUGHT TO LIGHT IN PELVIC OPERATIONS.

Robb's conclusions are the following:

1. In 323 out of 370 pelvic cases, no inflammatory changes in the appendix were found, even microscopically.
2. When a normal appendix is found in conjunction with disease of the pelvic organs, it is improbable that the latter has been brought about by a perforation of the appendix which has healed.
3. On the other hand, an old circumappendicitis and adhesions may often be looked upon as the result of a septic infection, originating in and spreading from the organs of generation.
4. An appendix which looks abnormal microscopically does not always show inflammatory changes on microscopical examination.
5. Nevertheless, when the removal of the appendix adds very little to the gravity of the abdominal operation, for the benefit of the patient it should be removed.
6. In the series of 370 cases there were four deaths, but careful analysis shows that the fatality could in no case be attributed to the removal of the appendix.—*American Jour. Obstetrics, August 1905.*

---

### A SIGN OVER A LITTLE COUNTRY STORE IN GEORGIA.

JONATHAN WILKINS.
Ice-cream in Season
and Embalming on Reasonable
Cash Terms.
Also Millinery and Tooth-
Pulling.
Boots, Shoes, Books and Bacon.
Coffins on the Instalment Plan.

—*Life.*

### GALL STONES.

Morison (*Edinburgh Monthly Journal*, October, 1905) notes the following facts : That gall stones may be present and cause in an uninflamed gall bladder no symptoms. Cholecystitis is the necessary irritant. If symptoms have once occurred they usually recur. Gall stone attacks are exceedingly painful, because when cholecystitis is present, the efforts of the gall bladder are to expel its contents. Serious symptoms are usually due to stones which are in the ducts. Repeated attempts on the part of the unstriated muscle of the gall bladder to expel its contents lead to its hypertrophy. If these attempts are unsuccessful inflammation and degeneration of the organ result, possibly gangrene and rupture, the latter resulting from tension. Pancreatitis may simulate gall stone disease and make a diagnosis impossible. The general condition in acute gall stone disease is that produced by acute septicœmia, and is caused by infectious germs. The gall bladder may contain bile, pus, or clear mucoid fluid. Spontaneous cures sometimes occur, but one is more likely to meet with fibroid thickening of the bladder, ulceration of the ducts, abscess of the liver, and other serious conditions. The treatment of this disease is surgical at the earliest possible moment.

---

### REASONS FOR REMOVING THE VERMIFORM APPENDIX.

Baker summarizes the history of twenty cases as follows:

1. The presence of adhesions of, or fecal concretions within, the appendix are not the only evidences of appendicitis which should influence the surgeon for its removal.

2. The appearance of a normal appendix at the time of the operation has proved to be unreliable, as three-fifths of the cases reported were suffering from chronic appendicitis.

3. The advantages to be derived as a prophylactic measure for the removal of the appendix, even in cases which prove normal, far outweigh the slight additional risk incurred by the operation.

4. The great frequency with which appendicular troubles present themselves warrant not only the removal of the appendix where it is easily accessible, when performing abdominal section for other lesions, but also the careful searching for and removal of it, even though it may appear normal from its gross appearance.—*American Jour. Obstetrics, August, 1902.*

---

### PARAVAGINAL OR ABDOMINAL OPERATION IN CARCINOMA OF THE UTERUS.

In a discussion on the relative merits of the two operative routes, G. Gellhorn (*St. Louis Medical Review,* June 3, 1905), comes to the following conclusions:

1. The radical abdominal operation, as far as the routine ablation of the lymphatic organs of the pelvis is concerned, has thus far failed to yield the desired results.

2. On the other hand, the eradication of the parametria has been found to reduce greatly the percentage of cures.

3. Consequently, the simple abdominal and vaginal operations which do not include this procedure must be discarded.

4. For the extirpation of the parametria, Wertheim's method is the best of the abdominal operations.

5. It has, however, certain advantages compared with the paravaginal method of Schuchardt.

6. Considering the encouraging results of igniextirpation, a combination of thermocautery with paravaginal extirpation gives promise of further improvement in operative results.

---

### MIDDLE EAR SUPPERATION.

Milligan (*British Medical Journal*, November 4th, 1905) summarizes the various modes of treatment which conduce to the rapid cure of acute septic otitis media, and prevent the onset of chronic disease as follows: Early and free incision of the swollen and bulging membrana tympani. Maintenance of free drainage through the incised membrane. Avoidance of inflation during the acute phases of inflammation of the mucosa lining the middle ear-cleft. Early removal of nasal and nasopharyngeal pathological entities. Early provision of drainage from the postero-external end of the middle ear-cleft in those cases where drainage through the membrana tympani has proved insufficient. Waggart states that with serous effusion medical treatment as a rule suffices. With mucopurulent catarrh, it is especially necessary to remove some causative factor in the nose or nasopharynx. In acute suppuration, besides the removal of the remote cause, pressure must be removed and the poison diluted without delay.

---

### A FURTHER CONTRIBUTION TO THE TREATMENT OF TYPHOID BACILLURIA WITH UROTROPIN.

In concluding a paper on this subject in the *Boston Medical and Surgical Journal* of August 17, 1905, Easton says:

Although urotropin may, in very rare cases, cause uncomfortable symptoms, this does not invalidate the use of the drug.

Urotropin is of least value in cases where an active inflammation of the bladder has occurred.

But, as far as this series of observations goes, the moderate use of urotropin throughout the disease prevents cystitis.

Finally, the routine administration of the drug in all cases of typhoid fever would seem to be strongly indicated, for by such a course of treatment bladder complications are avoided, the urine made innocuous to those brought in contact with the patient, and it is possible to discharge patients who have been sick with typhoid fever in full belief that, as far as the urine is concerned, they will be harmless to the community.

---

A NOTHNAGEL scholarship of $5,000 is announced to be established in memory of the late professor. The yearly income will be awarded as a prize for the best medical essay on such medical problems as may be proposed by the senate of the University of Vienna. Should no essay be satisfactory, the money is to be expended to encourage research on diseases of the intestines.

---

Individuals with bluish sclerotics, and with dark lanugo over the upper part of the back are usually of tuberculosis diathesis; and these signs are not inconsequential in making a diagnosis.—*American Journal of Surgery.*

### DIAGNOSIS OF SMALLPOX.

The confusing and conflicting opinions which prevail among physicians in the differentiation of smallpox and varicella are due to the variety of forms in which both of these diseases express themselves.

We are too apt to overlook the fact that smallpox and chicken-pox, like other diseases, are liable to present typical and atypical forms. This is most apt to occur when they coincidently invade the same field and commingle their operations. Each disease now assumes to a certain extent the livery of the other, which, in a measure, changes the characteristics of both, and often renders identification difficult. Divergent opinions will continue to confuse the medical mind until the causes of smallpox and varicella are definitely ascertained. The most experienced observers are yet divided on the question of relationship between these diseases. American authorities are inclined to ascribe them to separate and distinct forms of virus, while many English and German writers recognize varicella as a modified form of variola. Erasmus Wilson, Hebra, Kaposi and Kassowitz have adopted the latter theory, and their opinions are entitled to great weight; but the fact that chicken-pox often prevails where there is not a single case of smallpox to be found, and presents an entirely different history, affecting mostly children, while smallpox finds its most favorable soil for proliferation in the adult, weakens the position they have taken. It is possible their opinions have been formed from their observation of varicella as it develops in connection with smallpox. Here it assumes some of the outward forms of smallpox, but these forms do not mature. They do not produce the grave symptoms of smallpox. They complete their work in from eight to fourteen days, while smallpox requires four weeks or more. Chicken-pox differs also from varioloid in many respects. Varioloid passes through all the stages of variola, but they are milder in type and shorter in duration. Varicella runs its course, as above stated, in from one to two weeks; varioloid requires from three to four weeks, and variola from four to five weeks. The constancy of their separate histories would seem to bar a unity of origin in variola and varicella. We are too apt to base our opinion of chicken-pox on the common vesicular form in which it expresses itself, but this form is only constant when chicken-pox operates independently of smallpox. When it follows in the wake of smallpox, the initial lesion assumes the cone form and is described by some authorities as "varicella coniformis." It is this borrowed element of chicken-pox, with which we are not familiar, that often leads us to confound it with smallpox. If we bear this in mind we will have less trouble in distinguishing one from the other.

While it is difficult to recognize smallpox before the eruption appears, the following symptoms will lead us to prepare for its advent: A severe chill, persistent vomiting, pain in the loins of a marked character, and high fever. The appearance, in about three days after these symptoms have developed, of papules on the forehead, face, hands and wrists, gradually extending to other parts of the body, which inside of twenty-four hours are converted into umbilicated vesicles and later pass into pustules, will leave no doubt on the mind concerning the nature of the disease. In connection with these manifestations, if the fever has subsided after the appearance of the papular eruption and reappeared on the development of the pustular stage, our evidence of the disease is complete. It is readily distinguished from measles, scarlatina and syphilis; and we have only to bear in mind its different degrees of virulence, due to the potency of the virus and the sanitary environment of the patient, to differentiate it from varicella in all of its various forms.—*American Journal of Dermatology.*

---

After circumcision it is important to prevent adhesion of the reflected mucous fold of the prepuce to the corona glands by the daily passage of a probe about the corona, and by the use of vaseline.—*American Journal of Surgery.*

---

Surgical tuberculosis, no less than pulmonary tuberculosis, calls for the most careful general treatment, post-operative and otherwise.—*American Journal of Surgery.*

